# Research on the Visual Imagery of Posters Based on the Culture Code Theory of Design

**DOI:** 10.3389/fpsyg.2022.861366

**Published:** 2022-06-09

**Authors:** Luo Wen, Wang Jingjing, Wang Chen, Sun Luyu

**Affiliations:** School of Design, South China University of Technology, Guangzhou, China

**Keywords:** visual imagery, culture code theory of design, poster design, visual communication, semantic differential method

## Abstract

According to the Culture Code Theory of Design, posters can be divided into strategy aspect, meaning aspect, and technical aspect. This study explored the impact of visual imagery on poster communication and effect focus on the meaning and technical aspects which directly related to the audiences. The visual imagery preference of ten public poster samples is collected from 40 participants by online questionnaire with the semantic differential method. The results show that the audience’s visual imagery at the technical aspect is mainly related to the richness of colors, graphics, and texts. When receiving the information from the meaning aspect of the poster correctly, the visual imagery will be influenced by the type of theme. Most importantly, the same or similar visual imagery at the technical and meaning aspect encourages audiences to explore the deep information of posters. Poster works that keep visual imagery coherent and smooth at the two aspects can achieve a better effect.

## Introduction

Imagery, also known as psychological image, is the psychological feeling based on physical image and personal perception. Imagery can be both produced in the original consciousness field of the information producer, and extracted and formed after the audience receives the information (Tao, 2018). According to the source of sensation, imagery includes visual, auditory, olfactory, taste, and tactile imagery, among which visual imagery plays the most important role (Wu and Liu, 2016). The art psychology expert Rudolf Arnheim has proved from a large number of facts that whenever we think about a problem, there will be some imagery as the starting point or basis. In his view, creative thinking is inseparable from visual imagery in any cognitive field (Science and Art), and visual imagery is the bridge connecting science and art, rationality and sensibility (Zhencheng, 2005).

The poster is a functional visual design, which plays an important role in information communication and owns a significant relationship with visual imagery (Purnomo and Hidayatuloh, 2020). The visual imagery connection between designers and audiences influences the information exchange. According to the purpose, posters can be divided into four categories: public, political, cultural, and commercial posters. The main purpose of public posters is to spread the values of truth, goodness, and beauty to the public and serve the establishment of social morality. The main purpose of political posters is to spread political ideology. Cultural posters serve all kinds of cultural products and cultural exhibitions. Commercial posters are for-profit and serve enterprises and businesses (Yang, 2016). Compared with the other three categories, public posters have the widest audience and this research takes public posters as the main research object.

Current pieces of research mainly focus on the visual attraction and semantic understanding of posters. Most scholars believe that a good poster design needs to attract the audience’s attention at first. For example, colors and graphics with strong contrast will leave a deep visual impression on the audience, stimulate them to decode the information expressed by designers, and re-code it into personal cognition (Brown et al., 2013; Sentell, 2016). They apply visual dot-probe, eye tracker, semantic difference table to explore the impact of visual elements such as color, text, and composition of posters on the audience’s attention while ignoring the meaning communication of the poster (Wu and Liu, 2016; Yu and Ko, 2017; Chen et al., 2020; Molek-Kozakowska, 2020). On the other hand, although there are some research on the audience’s expression and emotion changes during viewing, the main methods include observation and interview. However, the observer’s subjective view easily to cause the omission and the misunderstanding of the audience’s feeling (Sentell, 2016; Charest et al., 2019). A combination of qualitative and quantitative research needs to be applied to the effect of posters.

**FIGURE 1:**
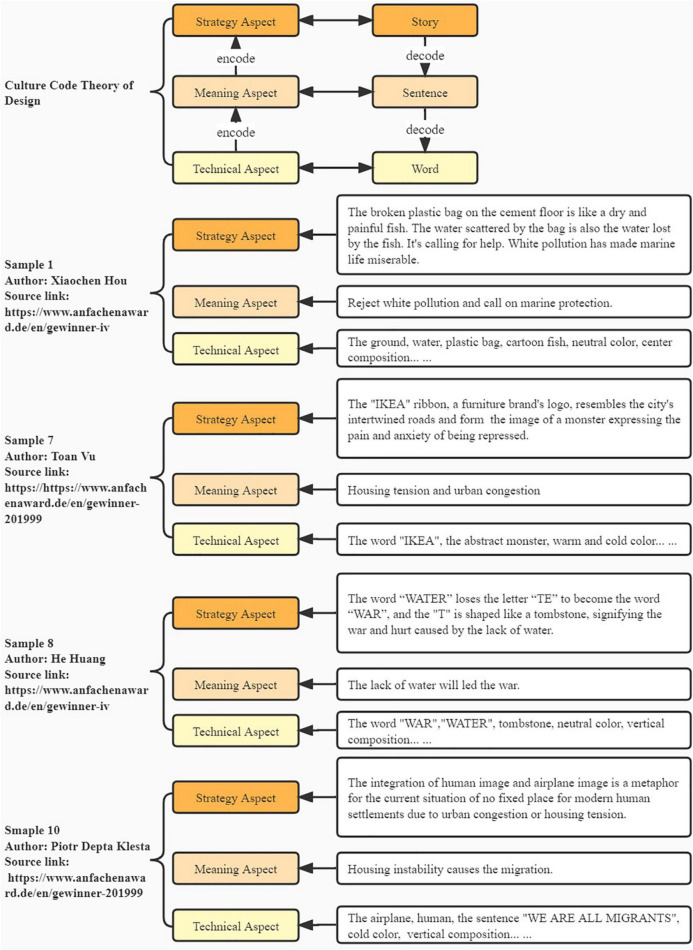
Culture code theory of design. (Poster source: https://www.anfachenaward.de/en).

As a culture system theory, the Culture Code Theory of Design was first proposed by Yufu Yang based on semiotic theory (Yufu, 2002). The Culture Code Theory of Design decomposes posters into three aspects: strategy, meaning, and technical aspect which is an effective tool for analyzing graphic design works. As shown in Figure 1, the strategy aspect is similar to the story structure in an article which is the designer’s ingenious thinking and is related to the designer’s cultural background. The meaning aspect can be compared to the words and sentences in an article. The technical aspect, also known as the element aspect, refers to all kinds of materials such as font, graphics, and colors as the single word forming a sentence. Take a work of the 4th German ANFACHEN Poster Design Award as an example shown in Figure 1. The broken plastic bag, ground, water, cartoon fish (technical aspect) are used to form a painful image of fish sending out the “HELP” signal on the dry ground (strategic aspect) to expresses the concern that white pollution has made marine life miserable, and calls on the public to protect marine resources (meaning aspect). In most cases, the strategy aspect will be deliberately hidden by the designer, which is difficult to grasp completely and accurately for audiences. The technical aspect and the meaning aspect are easier for audiences to receive and understand which is also the effect that the author hopes to achieve (Yufu, 2002; Yang et al., 2012). Therefore, this research mainly focuses on the meaning aspect and technical aspect of posters.

Poster design is an art design that requires both visual attraction and readability. Applying the Culture Code Theory of Design, we can completely study the visual imagery of posters from the technical aspect (color, text, graphics, etc.) and the meaning aspect (theme) to provide designers with design and creation strategies to improve the effect of poster design.

## Materials and Methods

### Poster Samples

The poster sample for this research comes from the German ANFACHEN Poster Design Award^1^. Every year, the 25 best posters will be awarded at a public exhibition with thousands of international posters. It has been held for four sessions since 2017 and involves four public themes: Woman, Tolerance, House and Living, and Water. A total of 100 winning works from the four sessions were selected as the initial sample set. An expert group composed of two design teachers and two design students coded 100 posters from the meaning aspect (theme) and the technical aspect (style, composition, graphics and text, and color) of the poster as shown in Table 1 (Zhang, 2015; Yang, 2016). Four feature sample sets are obtained by hierarchical clustering of SPSS (IBM SPSS Statistics 25, Armonk, NY, United States of America). The size of the posters in the four sample sets is 35, 24, 20, and 21, respectively. The final scale is 4:2:2:2 of the four clusters.

**TABLE 1:** The code rule of 10 poster samples.

Elements	Categories		
Theme	1 Woman; 4 Water	2 Tolerance;	3 House and Living;
Style	1 Abstract; 4 Cartoon;	2 Realism; 5 Text	3 Impressionism;
Composition	1 Central; 4 Vertical; 7 Surround;	2 Symmetrical; 5 Scattered; 8 Others	3 Horizontal; 6 Diagonal;
Tech	1 Repeat; 4 Gradient; 7 Texture;	2 Approximate; 5 Contrast; 8 Space;	3 Specific; 6 Emission; 9 Others
Content proportion	1 below 25%; 4 above 75%	2 25-50%;	3 50-75%;
Graphics	1 Concrete;	2 Semi-abstract;	3 Abstract;
Colors	1 Warm;	2 Cold;	3 Neutral

### Adjective Words

The poster evaluation text is collected from relevant literature, magazines, and websites. The Weiciyun website^2^ is used for word segmentation and word frequency statistics. A total of 54 adjectives are obtained. The expert group selects ten adjectives as the final evaluation basis for the poster work. Then, ten pairs of representative opposite words are chosen, including Soft-Intense, Popular-Individualized, Plain-Fashionable, Traditional-Modern, Elegant-Heroic, Serious-Lively, Cold-Enthusiastic, Static-Dynamic, Empty-Plump, Harmonious-Conflicting.

### Experiment Procedure

Forty participants are recruited in total. Half of the participants come from design majors, and the other half come from non-design majors, with 50% male and 50% female to eliminate the potential influence of gender and specialty on experiment results. All participants are informed of the experiment procedure and sign informed consent. This experiment has been approved by the Ethics Review Committee of the South China University of Technology.

Using the 7-level Likert scale and regarding the two words of each adjective pair as two extreme cases, the participants use the scale to choose the degree of personal feeling between each adjective pair. The poster samples appear completely on the computer screen or mobile screen at a specific time. All participants need to fill in the questionnaire twice. In the first questionnaire, the participants are reminded to complete the questionnaire for each poster sample based on first impressions. The first appearance time of the poster is 7 s according to the research on the first impression of online web pages (Kim and Fesenmaier, 2008). In the second questionnaire, the participants need to fill in the questionnaire after deep thinking and record their understanding of each poster sample so the appearance time depends on themselves.

## Results

First, the questionnaire with the same score of ten dimensions is considered invalid and is eliminated. There is an overall analysis of the visual imagery of the poster sample at the technical aspect and the meaning aspect with the factor analysis of SPSS (IBM SPSS Statistics 25, America). The factors with large loads in multiple factors are deleted (Static-Dynamic, Empty-Plump, Harmonious-Conflicting) and three principal factors are extracted from the seven factors. The final factor analysis result is shown in Table 2. The seven adjective pairs can be divided into three groups (KMO = 0.671, *p* < 0.001), and the interpretation rate of the total variance is 69.939%.

**TABLE 2:** Factor analysis.

Adjective	Factor 1	Factor 2	Factor 3
Traditional-Modern	0.788	0.041	0.015
Plain-Fashionable	0.744	0.241	0.081
Popular-Individualized	0.730	−0.015	0.292
Cold-Enthusiastic	0.100	0.871	0.055
Serious-Lively	0.089	0.868	0.087
Elegant-Heroic	−0.009	0.214	0.858
Soft-Intense	0.332	−0.063	0.775

**FIGURE 2:**
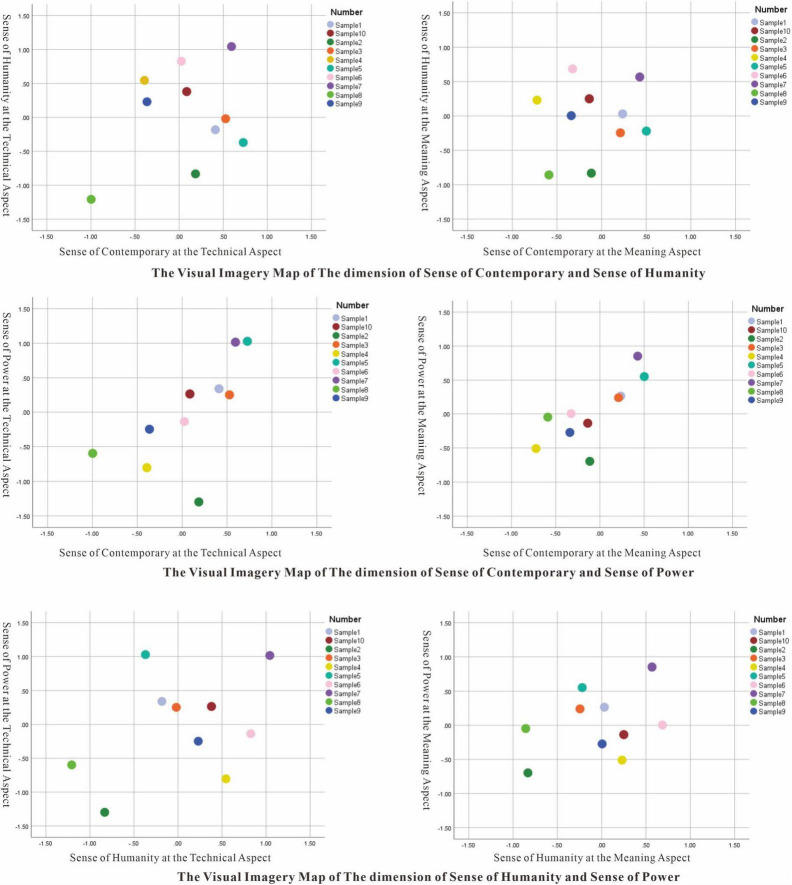
Visual imagery map of the technical aspect and the meaning aspect (Poster source: https://www.anfachenaward.de/en).

The evaluation criteria of the word pairs Traditional-Modern, Plain-Fashionable, and Popular-Individualized in Factor 1 is related to the social trend, so Factor 1 is named Sense of Contemporary. Factor 2 including Cold-Enthusiastic, Serious-Lively mainly describes the temperature of the poster, so Factor 2 is named Sense of Humanity. Factor 3 contains Elegant-Heroic and Soft-Intense that focus on the impact of the poster, so it is named Sense of Power. Take Factor 1, Factor 2, and Factor 3 as x, y, z coordinates, respectively, and the average of all samples in the three factors as the origin, the poster samples’ visual imagery maps of every two aspects are drawn according to the difference (Figure 2). Figure 2A is the visual imagery map of ten poster samples at the technical aspect and the meaning aspect with the Sense of Contemporary and the Sense of Humanity as the dimensions. Figure 2B is the visual imagery map in the dimensions of Sense of Contemporary and Sense of Power. Figure 2C is the visual imagery map in the dimensions of Sense of Humanity and Sense of Power. According to Figure 2, the sense of contemporary of samples 8 and 9 increase. The sense of humanity of samples 1, 5, and 8 increased. The sense of power of samples 2, 4, 6, and 8 increased. The biggest change occurred in sample 8 in three dimensions. In general, all samples show a trend of moving closer to the center in the meaning aspect.

The independent-samples *T* Test of SPSS (IBM) was used to explore the differences between gender and profession (*n* = 40). It showed that there is no significant difference in the questionnaire results between different genders. Only in the meaning aspect, the professional group compared with the non-professional group consider that sample 5 was more fashionable (*F* = 3.252, *p* < 0.05). Therefore, this research will ignore the influence of gender and specialty on visual imagery. Using the paired-samples *T* Test of SPSS (IBM), the results show that the meaning aspect makes sample 1 more dynamic and enthusiastic, sample 2 more modern and intense, sample 5 more plump, sample 6 more personalized and fashionable, and sample 8 more dynamic. The visual imagery of the other samples doesn’t change significantly.

## Discussion

The visual imagery at technical and meaning aspects of the poster is the emotional tendencies created by different colors, graphics, text, compositions, and themes. Current research shows that emphasizing emotion in design can help enhance the audience’s acceptance and understanding of design works (Kao and Du, 2020). The experiment results show that audiences have different emotional characteristics for different presentation methods and themes. In terms of the technical aspect, it is clear that the visual imagery of the poster samples with rich colors and graphics are intense and lively, while that of the posters with poor elements are soft and serious (Ji et al., 2020). As for the meaning aspect, deep thinking enhances the sense of humanity and the power of posters related to social issues such as housing tension, tolerance, and respect for women, which means the posters became colder and softer for the audience in a way that posters related to water themes do not. An interesting thing to be concerned about is that different expressions will lead to different changes in visual imagery. Both samples 2 and 8 are calling on the protection of water. Sample 1 selects the cartoon image of a painful fish, and sample 8 selects the neat words of “WAR” and the graphics of a tombstone. The meaning of the posters increases the sense of power of sample 8 but decreases the sense of power of sample 1.

In the detailed comparative analysis of the technical aspect and meaning aspect, there is no significant difference in the visual imagery of samples 4, 7, 9, and 10. Considering the higher understanding degree of samples 4 and 7 than that of samples 9 and 10, there may be two reasons to explain this situation. One reason is that the audience did not grasp the deep meaning of the poster sample so the meaning aspect does not affect the final visual imagery. The second reason is that the visual imagery of the meaning aspect and technical aspect is consistent, so the emotion would not change. The comparison between samples 7 and 9 can verify the two reasons. In sample 7, the IKEA banners are entwined to form a monster roaring which covers the full poster, and the graphics are abstract with blue and yellow color. The audience’s first feeling about the poster elements is conflict and strong which is consistent with the poster’s meaning of housing tension and urban crowding. Even if some audiences do not notice the hidden IKEA logo and the vague monster image in the picture, they can still accurately receive the expected message of the poster. In contrast, sample 9 also expresses the theme Housing and Living where the text “We are all migrants” assists the semi-abstract graphics like a human-shaped airplane to express the housing crisis and housing instability. The visual imagery of the technical aspect with blue, white, and black color and the central composition conflicts with the strong visual imagery of the meaning layer about human existence. Even with the help of text, the understanding of the meaning of sample 9 is not better than that of sample 7.

It can be seen that only when the audience receives the information correctly, the meaning aspect affects the visual imagery of the technical aspect. And the poster with unified visual imagery at both aspects has a higher understanding. The consistency between the first impression and deep thinking, from the technical aspect to the meaning aspect plays a vital role in the persuasive effect for poster design.

## Conclusion

This research found that different visual imagery will be produced by different colors, compositions, and themes of the poster. The audience will have a higher understanding of the poster that owns consistent visual imagery in the viewing process. The result suggests that the posters with the theme of women and children will awaken the softness of the audience, which can be assisted by softer colors and stable composition. The strong and conflicting visual effect will be more conducive to the expression of social themes such as environmental protection and social competition.

In conclusion, the same or similar visual imagery at the technical aspect and the meaning aspect encourage audiences to explore and think further. The posters that keep the visual imagery coherent can achieve a better effect.

## Data Availability Statement

The original contributions presented in the study are included in the article/Supplementary Material, further inquiries can be directed to the corresponding author/s.

## Ethics Statement

The studies involving human participants were reviewed and approved by Ethics Review Committee of South China University of Technology. The patients/participants provided their written informed consent to participate in this study.

## Author Contributions

LW contributed to the conception of the study. WJ performed the experiment and the data analyses and wrote the manuscript. WC contributed significantly to manuscript preparation. SL helped perform the analysis with constructive discussions. All authors contributed to the article and approved the submitted version.

## Conflict of Interest

The authors declare that the research was conducted in the absence of any commercial or financial relationships that could be construed as a potential conflict of interest.

## Publisher’s Note

All claims expressed in this article are solely those of the authors and do not necessarily represent those of their affiliated organizations, or those of the publisher, the editors and the reviewers. Any product that may be evaluated in this article, or claim that may be made by its manufacturer, is not guaranteed or endorsed by the publisher.

## Footnotes

1
https://www.anfachenaward.de/en

2
www.weiciyun.com
